# Periphery Biomarkers for Objective Diagnosis of Cognitive Decline in Type 2 Diabetes Patients

**DOI:** 10.3389/fcell.2021.752753

**Published:** 2021-10-20

**Authors:** Yanchao Liu, Shujuan Zhang, Benrong He, Liangkai Chen, Dan Ke, Shi Zhao, Yao Zhang, Wei Wei, Zhipeng Xu, Zihui Xu, Ying Yin, Wen Mo, Yanni Li, Yang Gao, Shihong Li, Weijin Wang, Huiling Yu, Dongqin Wu, Guilin Pi, Tao Jiang, Mingmin Deng, Rui Xiong, Huiyang Lei, Na Tian, Ting He, Fei Sun, Qiuzhi Zhou, Xin Wang, Jinwang Ye, Mengzhu Li, Nan Hu, Guoda Song, Wenju Peng, Chenghong Zheng, Huaqiu Zhang, Jian-Zhi Wang

**Affiliations:** ^1^Ministry of Education Key Laboratory for Neurological Disorders, Hubei Key Laboratory for Neurological Disorders, Department of Pathophysiology, School of Basic Medicine, Tongji Medical College, Huazhong University of Science and Technology, Wuhan, China; ^2^Department of Neurosurgery, Tongji Medical College, Tongji Hospital, Huazhong University of Science and Technology, Wuhan, China; ^3^Department of Nutrition and Food Hygiene, Hubei Key Laboratory of Food Nutrition and Safety, Ministry of Education Key Laboratory of Environment and Health, School of Public Health, Tongji Medical College, Huazhong University of Science and Technology, Wuhan, China; ^4^Department of Endocrinology, Central Hospital of Wuhan, Wuhan, China; ^5^Liyuan Hospital, Tongji Medical College, Huazhong University of Science and Technology, Wuhan, China; ^6^Department of Neurology, Zhongnan Hospital of Wuhan University, Wuhan, China; ^7^Health Service Center of Jianghan District, Wuhan, China; ^8^Department of Endocrinology, Wuhan Hospital of Traditional Chinese Medicine, Wuhan, China; ^9^Co-innovation Center of Neuroregeneration, Nantong University, Nantong, China

**Keywords:** type 2 diabetes mellitus, Alzheimer’s disease, mild cognitive impairment, plasma β-amyloid, platelet glycogen synthase kinase-3β

## Abstract

**Introduction:** Type 2 diabetes mellitus (T2DM) is an independent risk factor of Alzheimer’s disease (AD), and populations with mild cognitive impairment (MCI) have high incidence to suffer from AD. Therefore, discerning who may be more vulnerable to MCI, among the increasing T2DM populations, is important for early intervention and eventually decreasing the prevalence rate of AD. This study was to explore whether the change of plasma β-amyloid (Aβ) could be a biomarker to distinguish MCI (T2DM-MCI) from non-MCI (T2DM-nMCI) in T2DM patients.

**Methods:** Eight hundred fifty-two T2DM patients collected from five medical centers were assigned randomly to training and validation cohorts. Plasma Aβ, platelet glycogen synthase kinase-3β (GSK-3β), apolipoprotein E (ApoE) genotypes, and olfactory and cognitive functions were measured by ELISA, dot blot, RT-PCR, Connecticut Chemosensory Clinical Research Center (CCCRC) olfactory test based on the diluted butanol, and Minimum Mental State Examination (MMSE) test, respectively, and multivariate logistic regression analyses were applied.

**Results:** Elevation of plasma Aβ1-42/Aβ1-40 is an independent risk factor of MCI in T2DM patients. Although using Aβ1-42/Aβ1-40 alone only reached an AUC of 0.631 for MCI diagnosis, addition of the elevated Aβ1-42/Aβ1-40 to our previous model (i.e., activated platelet GSK-3β, ApoE ε4 genotype, olfactory decline, and aging) significantly increased the discriminating efficiency of T2DM-MCI from T2DM-nMCI, with an AUC of 0.846 (95% CI: 0.794–0.897) to 0.869 (95% CI: 0.822–0.916) in the training cohort and an AUC of 0.848 (95% CI: 0.815–0.882) to 0.867 (95% CI: 0.835–0.899) in the validation cohort, respectively.

**Conclusion:** A combination of the elevated plasma Aβ1-42/Aβ1-40 with activated platelet GSK-3β, ApoE ε4 genotype, olfactory decline, and aging could efficiently diagnose MCI in T2DM patients. Further longitudinal studies may consummate the model for early prediction of AD.

## Introduction

Alzheimer’s disease (AD) is the most common cause of dementia in the elderly. With the worldwide population aging, the prevalence of AD is increasing. The currently recognized strategies for the clinical diagnosis of AD mainly include (1) brain imaging such as positron emission tomography (PET) for measuring brain amyloidosis and tauopathies, magnetic resonance imaging (MRI) for measuring medial temporal atrophy, and fluorodeoxyglucose positron emission tomography (FDG-PET) for measuring low glucose metabolism; (2) measurement of cerebrospinal fluid (CSF) biomarkers, such as Aβ42 and phospho-tau levels (Bocchetta et al., 2015); and (3) different types of neuropsychiatric tests, such as Mini Mental State Examination (MMSE). These methods are either expensive or invasive or subjective, which makes them hardly acceptable by the patients, and the diagnoses made by different doctors are not comparable. Thus, there is an urgent need to find peripheral biomarkers for a non-expensive, non-invasive, and objective diagnosis of AD in the early stage. Generally, it is already too late to receive an effective intervention by the time a patient sees the doctor with a memory complaint. Therefore, focusing on high-risk factors of AD, such as aging and type 2 diabetes mellitus (T2DM), may be helpful.

Type 2 diabetes mellitus is an independent risk factor of AD, and T2DM patients show a significantly increased prevalence of AD compared with those without diabetes (Xu et al., 2004). Evidence suggests that patients with T2DM and AD share common genetic background, environmental risk factors, and underlying pathologies (de Matos et al., 2018). T2DM contributes to cognitive decline and brain atrophy (Callisaya et al., 2019; Moran et al., 2019). Similar to that of AD, the incidence of T2DM is also rapidly increasing with population aging. Therefore, finding peripheral biomarkers to identify in the T2DM populations who may develop into MCI/AD and thus to design specific interventions should be promising to hold back the progression of diabetes into AD and eventually to reduce the prevalence of AD.

Peripheral blood has been widely employed for biomarker studies. For instance, it has been reported that glycogen synthase kinase-3β (GSK-3β), a serine/threonine kinase, is increased in the periphery white blood cells of AD and MCI patients (Hye et al., 2005), and activation of GSK-3β promotes tau hyperphosphorylation (Plattner et al., 2006) and Aβ toxicity (Yi et al., 2018). Lithium chloride inhibits GSK-3β and decreases tau phosphorylation (Tajes et al., 2008). A meta-analysis suggests that olfactory dysfunction can predict the incidence of MCI (Rahayel et al., 2012). The polymorphism of apolipoprotein E (ApoE) alleles is involved in late-onset AD, and the individuals carrying the ε4 allele have an increased risk of AD compared with those carrying ε3 and ε2 alleles (Liu et al., 2013). We recently reported that platelet GSK-3β activation, olfactory deficit, ApoE ε4 genotype, and aging were associated with MCI in T2DM patients (Xu et al., 2016).

Both T2DM and AD are common age-related amyloid diseases (Umegaki, 2014). In cognitively impaired hemodialysis patients, plasma Aβ42 level is associated with cognitive performance, and inducing peripheral Aβ sink by hemodialysis is considered as an anti-amyloid strategy (Tholen et al., 2016). Regarding the changes of Aβ levels in CSF and periphery blood, contradictory results were reported in AD (Mayeux et al., 1999, 2003; Irizarry, 2004; Pomara et al., 2005; van Oijen et al., 2006; Graff-Radford et al., 2007; Blennow et al., 2010; Cosentino et al., 2010; Buchhave et al., 2012). To date, it is not known how the plasma Aβ level is altered in T2DM patients and whether the change of the plasma Aβ level is associated with the cognitive decline in T2DM patients.

In the present study, we first measured the levels of Aβ1-40 and Aβ1-42 in T2DM patients with (T2DM-MCI) or without MCI (T2DM-nMCI). We found that the level of plasma Aβ1-42 was significantly increased in T2DM-MCI compared with that in T2DM-nMCI. By multivariate logistic regression analyses of the data received from both current and our previous cohorts, we observed that an elevated ratio of Aβ1-42 to Aβ1-40 was an independent risk factor for cognitive decline in T2DM patients; furthermore, addition of the elevated Aβ1-42/Aβ1-40 into our previously established model (i.e., upregulated platelet GSK-3β activity, ApoE ε4 genotype, olfactory dysfunction, and aging) remarkably increased the diagnostic efficiency of MCI from T2DM patients in both training and validation cohorts.

## Materials and Methods

### Study Population and Inclusion/Exclusion Criteria

This study had a multicenter case–control design, including 852 middle-aged or elder T2DM patients recruited from January 2012 to November 2018 from five medical centers in Wuhan, China. The inclusion criteria were as follows: (1) age ≥ 50 years, (2) long-term residence (≥5 years), and (3) ability to complete the neuropsychological test and write informed consent. The exclusion criteria were as follows: (1) with a history of head trauma, stroke, brain tumor, coma, transient ischemic attack, epilepsy, and other central nervous system diseases that could cause dementia or presence of dementia before T2DM; (2) auditory/visual disorders; (3) thyroid disease; (4) possible or known drugs affecting cognitive function abuse; (5) alcohol addiction; (6) diagnosed depression; and (7) schizophrenia and other psychiatric disorders, such as acute stress disorder, post-traumatic stress disorder, and acute transient psychosis. The patients were divided into training and verification cohorts based on the recruiting time and the hospitals from which the patients were recruited.

### Ethics and Informed Consent

The study protocol was approved by the Medical Ethics Committee, Tongji Medical College, Huazhong University of Science and Technology in accordance with the principles of the Helsinki Declaration II. All participants had provided written informed consent. Our study’s biosecurity and safety procedures adhered to the “Regulations on Safety Management of Laboratory Technology of Huazhong University of Science and Technology.”

### Mild Cognitive Impairment Diagnosis Criteria

All patients underwent conventional medical history and physical examinations. The MMSE test was conducted by two of our experienced inspectors with neurology training. MCI was diagnosed based on Petersen’s criteria: (1) memory complaint, (2) normal activities of daily living, (3) normal general cognitive function, (4) the abnormal memory ability for the age, (5) no dementia, and (6) MMSE scores at 24–27.

### Biomarker Measurement

#### Peripheral Blood Isolation and Storage

For biomarker measurement, fresh blood in an EDTA K2 anticoagulant tube was centrifuged for 15 min at 60 × *g* at 4°C in a low-speed centrifuge. The blood was divided into three layers, namely, plasma (including platelet), white blood cells, and red blood cells from top to bottom. The upper layer of plasma was further centrifuged at 1,500–2,000 × *g* at 4°C for 15 min, and the supernatant was collected as pure plasma without platelet. The blood samples were separated within 2 h, and the aliquots of the three fractions were stored at −80°C before use.

#### ELISA for Plasma Aβ1-40 and Aβ1-42 Measurements

The plasma Aβ1-40 and Aβ1-42 were detected by using a commercial ELISA kit by following the manufacturer’s instruction (E-EL-H0542c and E-EL-H0543c, Elabscience, China). Specifically, the Aβ1-40 and Aβ1-42 standards, or vehicle as blank, or samples (100 μl each) were diluted and added into 96-well plates in duplicates, and the plates were incubated for 90 min at 37°C. After decanting the liquid from each well without washing, 100 μl of biotinylated detection antibody working solution was immediately added to each well and incubated for 1 h at 37°C. The solution was decanted from each well, and 350 μl of washing buffer was added to each well three times. Then a horseradish peroxidase (HRP)-conjugated working solution (100 μl each) was added to each well and incubated for 30 min at 37°C. The plate was repeatedly washed five times. Then 90 μl of the substrate reagent was added to each well and incubated for about 15 min at 37°C in the dark. Finally, a stop solution (100 μl each) was added to each well, and the optical density (OD value) was recorded at once with a microplate reader set to 450 nm.

#### Connecticut Chemosensory Clinical Research Center Test for Olfactory Function

The olfactory score was measured using the Connecticut Chemosensory Clinical Research Center (CCCRC) test based on the diluted times of butanol. The butanol was diluted into 12-grade concentrations (2.3 × 10^–5^%–4%). The participants were exposed to the flasks from low to high concentrations of butanol along with a blank and were asked to identify whether they smelled anything and which one smelled stronger. A higher score during the CCCRC test indicates a more severe olfactory impairment.

#### Apolipoprotein E Genotype Measurement

For ApoE genotype detection, the blood DNA extraction kit from Omega Bio-Tek was used to extract DNA from the isolated white blood cells by following the manufacturer’s instruction. To help improve DNA yield, the column was incubated for 5 min after being added with the elution buffer; for increasing the yield while maintaining the elution volume, the eluent collected from the first elution was repeatedly applied. The prepared DNA was stored in a −20°C before use.

Gerard’s method with modifications in the multiplex amplification refractory mutation system PCR was applied for ApoE genotyping (Donohoe et al., 1999). The primers were designed and synthesized by Invitrogen Life Technologies (Shanghai, China). Primer sequences were as described in the previous study (Xu et al., 2016). SNP genotyping was applied by Tianyi Huiyuan Technologies (Wuhan, China).

#### Dot Blot for Glycogen Synthase Kinase-3β Assay

The total GSK-3β and GSK-3β-S9 (serine-9 phosphorylated GSK-3β, the inactive form of the kinase; and the ratio of GSK-3β-Total/GSK-3β-S9, as a measure of GSK-3β activity) in the platelet were measured by dot blot and normalized by the same control (Xu et al., 2016). Advanced Tyrode’s solution and 1x PBS was used to purify and wash platelets. The platelet samples were diluted to the same concentration (7.5 μg/μl), and 2 μl each was evenly sampled on the nitrocellulose membrane. After air-drying at room temperature, the membrane was blocked with 5% skim milk powder. The primary antibody was incubated at 4°C overnight, and the secondary antibody was incubated at room temperature for 1 h. After TBS-T cleaning, the membrane was scanned and analyzed by Odyssey. All measurements were performed in a blind manner.

### Covariates of the Study Population

Demographic and health information, including age, sex, family history of diabetes, diabetes duration, insulin treatment, and diabetic complications, were collected using a semi-structured questionnaire. History of diseases, including hypertension, hyperlipidemia, and coronary heart disease (CHD), was determined by medical records. Hemoglobin A1c (HbA1c) was measured using fasting serum through venipuncture.

### Statistical Analysis

The differences in plasma Aβ1-42/Aβ1-40, platelet GSK-3β activity presented by an increased ratio of total GSK-3β to serine-9 phosphorylated GSK-3β (rGSK-3β), and basic characteristics between patients and controls were assessed using the Kruskal–Wallis test (continuous variables and skewed distribution), Student’s *t*-test (continuous variables and normal distribution), and χ^2^ test (categorical variables).

Multivariable logistic regression analysis was performed to estimate the independent association between plasma Aβ1-42/Aβ1-40, platelet rGSK-3β, and MCI among T2DM patients in two independent cohorts. The plasma Aβ1-42/Aβ1-40 and platelet rGSK-3β levels were categorized into quartiles based upon their distribution in the control subjects to calculate the odds ratios (OR) and 95% confidence intervals (CIs). The crude analysis included only plasma Aβ1-42/Aβ1-40 without any adjustment. The subsequent analysis was adjusted for potential confounders, including sociodemographic factors, lifestyle habits, medical histories, and laboratory characteristics. The linear trend was tested by using the median value of each quartile as a continuous variable in the models.

By combining the data received in the present study and those in our previous study (Xu et al., 2016), five variables [age, ApoE ε4, olfactory score, plasma Aβ1-42/Aβ1-40, and platelet rGSK-3β (Total/S9)] were selected for establishing the diagnostic model. To confirm the model, we examined the associations of each clinical parameter and biomarker with MCI by applying univariate logistic regression analysis and calculated Akaike’s information criterion (AIC) for each variable. The established models were validated with respect to their discrimination and calibration ability (Alba et al., 2017). The receiver operating characteristic curve (ROC) analysis was used to calculate the area under the curve (AUC) and evaluate the diagnostic discrimination of the proposed model. The AUC with a 95% CI was computed by using 1,000 bootstrap resampling (Steyerberg et al., 2003). The agreement between the diagnosed probability and the actual outcome was evaluated by calibration plotting using 1,000 bootstraps. The diagnosing performance between the current model and our previous model (Xu et al., 2016) was compared using C-statistics and net reclassification index (NRI); the latter was calculated using the continuous approach (Pencina et al., 2011) with 1,000 bootstrap replications to estimate the 95% CI (implemented in the R package nricens).

All analyses were performed using SPSS 26.0 IBM software, the R software (The R Foundation^1^, version 3.5.0), and Empower Stats (X&Y Solutions, Inc., Boston, MA, United States)^2^.

## Results

### Baseline Characteristics of the Study Population in the Training Cohort and the Validation Cohort

The demographic and clinical data of the participants in the training cohort and the validation cohort are shown in Table 1. Compared with T2DM-nMCI subjects, the T2DM-MCI subjects had older age, higher percentage of having an ApoE ε4 allele (heterozygous), higher level of plasma Aβ1-42/Aβ1-40 ratio, higher platelet GSK-3β activity, and higher olfactory score that indicated decline of olfactory function (Table 1).

**TABLE 1:** Baseline characteristics and potential biomarkers of T2DM patients in the training cohort and the validation cohort.

	Training cohort		*p*-value	Validation cohort		*p*-value
			
	T2DM-nMCI (*n* = 274)	T2DM-MCI (*n* = 89)		T2DM-nMCI (*n* = 225)	T2DM-MCI (*n* = 264)	
Age (years)	62.06 (7.38)	66.47 (9.36)	<0.001	65.84 (6.32)	68.15 (8.05)	<0.001
Male, *n* (%)	109 (39.78%)	24 (26.97%)	0.029	96 (42.67%)	102 (38.64%)	0.366
Hypertension, *n* (%)	150 (54.74%)	40 (44.94%)	0.108	136 (60.44%)	169 (64.02%)	0.417
Hyperlipidemia, *n* (%)	22 (8.03%)	6 (6.74%)	0.692	74 (32.89%)	89 (33.71%)	0.847
CHD, *n* (%)	31 (11.31%)	10 (11.24%)	0.984	28 (12.44%)	32 (12.12%)	0.914
Family history of diabetes, *n* (%)	28 (12.44%)	32 (12.12%)	0.579	75 (33.33%)	83 (31.44%)	0.655
Diabetes duration (years)	7.00 (3.00–11.00)	8.00 (4.00–10.00)	0.224	7.00 (2.00–13.00)	7.00 (2.00–12.00)	0.931
Insulin treatment, *n* (%)	126 (45.99%)	35 (39.33%)	0.27	107 (47.56%)	101 (38.26%)	0.04
Diabetic complications, *n* (%)	165 (60.22%)	52 (58.43%)	0.77	121 (53.78%)	141 (53.41%)	0.94
HbA1c	8.12 (1.97)	8.02 (1.92)	0.765	7.35 (2.77)	7.75 (2.85)	0.13
APOE ε2 (+), *n* (%)	47 (17.15%)	14 (15.73%)	0.76	37 (16.44%)	49 (18.56%)	0.54
APOE ε3 (+), *n* (%)	263 (95.99%)	80 (89.89%)	0.06	221 (98.22%)	253 (95.83%)	0.19
APOE ε4 (+), *n* (%)	29 (10.58%)	18 (20.22%)	0.019	29 (12.89%)	62 (23.48%)	0.003
Olfactory score	6.51 (1.39)	7.94 (1.90)	<0.001	7.07 (1.48)	7.95 (1.48)	<0.001
GSK-3β (total)	0.82 (0.38–1.56)	0.84 (0.31–1.76)	0.862	1.12 (0.75–1.56)	1.89 (1.40–2.60)	<0.001
GSK-3β (S9)	1.57 (0.73–2.47)	0.68 (0.18–1.78)	<0.001	1.91 (1.16–2.77)	1.46 (0.96–2.22)	<0.001
rGSK-3β (Total/S9)	0.70 (0.37–0.95)	1.43 (0.79–2.34)	<0.001	0.56 (0.43–0.86)	1.21 (0.93–1.67)	<0.001
Aβ1-40	155.11 (108.88–204.01)	134.49 (102.07–178.04)	0.025	205.97 (151.35–270.97)	179.74 (92.42–269.55)	0.007
Aβ1-42	56.47 (39.03–100.04)	77.75 (48.06–112.62)	0.003	64.68 (47.44–79.75)	69.35 (54.23–93.44)	<0.001
Aβ1-42/Aβ1-40	0.43 (0.24–0.75)	0.63 (0.39–0.93)	<0.001	0.32 (0.20–0.50)	0.41 (0.27–0.89)	<0.001
MMSE	29.09 (0.84)	25.35 (1.88)	<0.001	28.64 (0.71)	24.55 (2.86)	<0.001

*Data were presented as mean (SD), *n* (%), or median (interquartile range).*

*T2DM, type 2 diabetes mellitus; MCI, mild cognitive impairment; T2DM-nMCI, T2DM without MCI group; T2DM-MCI, T2DM with MCI group; CHD, coronary heart disease; HbA1c, hemoglobin A1c; ApoE, apolipoprotein E; GSK-3β, glycogen synthase kinase-3β; rGSK-3β, ratio of GSK-3β-Total/GSK-3β-S9; MMSE, the minimum mental state examination.*

### Elevated Plasma Aβ1-42/Aβ1-40 Could Discriminate T2DM-MCI From T2DM-nMCI

We first measured whether the levels of Aβ1-40 and Aβ1-42 were different in T2DM-MCI and T2DM-nMCI groups. We observed that the level of Aβ1-42 was significantly increased while that of Aβ1-40 was decreased, resulting in an increased ratio of Aβ1-42/Aβ1-40 in T2DM-MCI patients compared with that in the T2DM-nMCI group (Table 1). Then, we applied the ROC model to calculate the sensitivity, specificity, accuracy, and AUC of different types of plasma Aβ in the training cohort (Table 2). Plasma Aβ1-40 showed a maximal AUC of 0.579 and an accuracy of 51.8%. Similarly, the diagnostic value of plasma Aβ1-42 for MCI revealed an AUC of 0.605 and an accuracy of 52.3% against non-MCI. An AUC of 0.631 and an accuracy of 61.4% for MCI were shown when the ratio of plasma Aβ1-42/Aβ1-40 was used. Therefore, using the elevated Aβ1-42/Aβ1-40 ratio was better than using the changed Aβ1-40 or Aβ1-42 alone or a pairwise combination in diagnosing MCI.

**TABLE 2:** Efficacy of different types of plasma Aβ in diagnosing MCI in the training cohort of T2DM patients.

Variables	Sensitivity	Specificity	Accuracy	AUC (95% CI)	Cutoff
Aβ1-40	0.685	0.464	0.518	0.579 (0.513–0.645)	159.81
Aβ1-42	0.708	0.464	0.523	0.605 (0.541–0.669)	53.23
Aβ1-42/Aβ1-40	0.596	0.620	0.614	0.631 (0.567–0.696)	0.54
Aβ1-40 + Aβ1-42	0.753	0.442	0.518	0.626 (0.561–0.692)	
Aβ1-40 + Aβ1-42/Aβ1-40	0.809	0.409	0.507	0.635 (0.570–0.700)	
Aβ1-42 + Aβ1-42/Aβ1-40	0.607	0.609	0.609	0.632 (0.567–0.696)	
Aβ1-40 + Aβ1-42 + Aβ1-42/Aβ1-40	0.584	0.628	0.617	0.632 (0.567–0.698)	

*ROC, receiver operating characteristics; AUC, area under curve; CI, confidence interval.*

**FIGURE 1:**
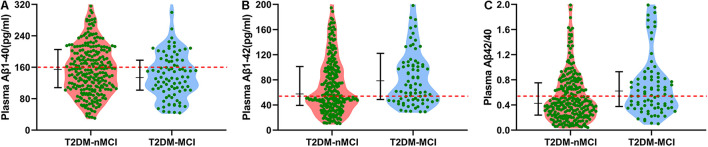
Cutoff points of different types of plasma Aβ in discriminating T2DM-MCI from T2DM-nMCI in the training cohort. **(A)** By using Aβ1-40, most of the population in T2DM-nMCI and T2DM-MCI groups was distributed under the cutoff point (159.81 pg/ml; red line). **(B)** By using Aβ1-42, most of the population in T2DM-nMCI and T2DM-MCI groups was distributed above the cutoff point (53.23 pg/ml; red line). **(C)** By using Aβ1-42/Aβ1-40, the T2DM-MCI could be nicely discriminated from T2DM-nMCI patients at cutoff 0.54. Data were presented as median (interquartile range).

To maximize the diagnostic efficiency of plasma Aβ, we further applied the best cutoff point, i.e., the maximal AUC in the ROC curve, to evaluate the population distribution in T2DM-nMCI and T2DM-MCI groups (Figure 1). For each ROC, the best cutoff point was determined by using Youden’s index, which optimized biomarker performance when equal weight was given to sensitivity and specificity and represented the likelihood of a positive test result in subjects with the condition versus those without the condition (Perkins and Schisterman, 2005). We found that the ratio of plasma Aβ1-42/Aβ1-40 at the cutoff of 0.54 but not at the level of Aβ1-40 or Aβ1-42 alone could maximally discriminate MCI from n-MCI (Table 2). These data confirmed that only the increased plasma Aβ1-42/Aβ1-40 ratio but not Aβ1-40 or Aβ1-42 alone could discriminate T2DM-MCI from T2DM-nMCI patients.

### Elevated Plasma Aβ1-42/Aβ1-40 Could Serve as an Independent Risk Factor for Mild Cognitive Impairment in Type 2 Diabetes Mellitus Patients Analyzed by Using Multivariate Logistic Regression Analysis

To explore whether the increased ratio of plasma Aβ1-42/Aβ1-40 could be an independent risk factor for diagnosing MCI in T2DM patients, we used multivariate logistic regression analysis. The ORs and 95% CIs for MCI were 1.00 (ref.), 2.22 (0.95–5.22), 2.98 (1.30–6.82), and 3.78 (1.69–8.47), with *p* trend = 0.002 and with the increase in quartile of the plasma Aβ1-42/Aβ1-40 in the training cohort (Table 3). After adjustment for age, sex, hypertension, hyperlipidemia, CHD, family history of diabetes, diabetes duration, HbA1c, insulin treatment, and diabetic complications, no significant change was observed: the ORs and 95% CIs for MCI were 1.00 (ref.), 2.32 (0.95–5.65), 2.80 (1.18–6.67), 3.53 (1.52–8.19), and 2.13 (1.39–3.28), with *p* trend = 0.007. These data excluded the contribution of the above-mentioned variables for the cognitive decline in T2DM patients. On the other hand, the association between plasma Aβ1-42/Aβ1-40 and MCI was remarkably strengthened after being adjusted by the previously identified biomarkers, including rGSK-3β, ApoE ε4, and olfactory score: ORs (95% CIs) of 1.00 (ref.), 3.14 (1.01–9.79), 5.33 (1.69–16.78), and 6.52 (2.16–19.69), with *p* trend = 0.001 in the training cohort and ORs (95% CIs) of 1.00 (ref.), 1.09 (0.55–2.17), 1.15 (0.58–2.28), and 2.50 (1.31-4.74), with *p* trend <0.001 in the validation cohort (Table 3). In the fully adjusted model, with a 1-value increment of plasma Aβ1-42/Aβ1-40, the ORs for MCI were 3.15 (95% CI: 1.80–5.52) in the training cohort and 2.40 (95% CI: 1.62–3.55) in the validation cohort (Table 3). These data together indicated that an increased plasma Aβ1-42/Aβ1-40 was an independent risk factor for MCI in middle-aged and elderly patients with T2DM.

**TABLE 3:** Efficacy of the elevated plasma Aβ1-42/Aβ1-40 ratio in diagnosing MCI in both training and validation cohorts of T2DM patients.

	Quartile of the plasma Aβ1-42/Aβ1-40					
	
	Q1 (Lowest)	Q2	Q3	Q4 (Highest)	Increase 1 value of Aβ1-42/Aβ1-40	*p* trend*
**Training cohort**						
Aβ1-42/Aβ1-40 levels	<0.247	0.247–0.434	0.434–0.754	≥0.754		
Cases/controls, n	9/69	20/69	26/67	34/69		
Aβ1-42/Aβ1-40, OR	1.00 (ref.)	2.22 (0.95–5.22)	2.98 (1.30–6.82)	3.78 (1.69–8.47)	2.14 (1.45–3.15)	0.002
Aβ1-42/Aβ1-40-Adjusted-1, OR	1.00 (ref.)	2.32 (0.95–5.65)	2.80 (1.18–6.67)	3.53 (1.52–8.19)	2.13 (1.39–3.28)	0.007
Aβ1-42/Aβ1-40-Adjusted-2, OR	1.00 (ref.)	3.14 (1.01–9.79)	5.33 (1.69–16.78)	6.52 (2.16–19.69)	3.15 (1.80–5.52)	0.001
**Validation cohort**						
Aβ1-42/Aβ1-40 levels	<0.203	0.203–0.320	0.320–0.497	≥0.497		
Cases/controls, n	42/56	51/56	62/57	109/56		
Aβ1-42/Aβ1-40, OR	1.00 (ref.)	1.21 (0.70–2.11)	1.45 (0.85–2.48)	2.60 (1.55–4.34)	2.22 (1.56–3.15)	<0.001
Aβ1-42/Aβ1-40-Adjusted-1, OR	1.00 (ref.)	1.02 (0.57–1.82)	1.64 (0.93–2.89)	3.15 (1.83–5.43)	2.64 (1.80–3.89)	<0.001
Aβ1-42/Aβ1-40-Adjusted-2, OR	1.00 (ref.)	1.09 (0.55–2.17)	1.15 (0.58–2.28)	2.50 (1.31–4.74)	2.40 (1.62–3.55)	<0.001

*OR, odds ratio (95% CI).*

*Aβ1-42/Aβ1-40-Adjusted-1 means the Aβ ratio was adjusted for age (in continuous), sex, hypertension (yes/no), hyperlipidemia (yes/no), CHD (yes/no), family history of diabetes (yes/no), diabetes duration (in continuous and quadratic terms), HbA1c (in continuous and quadratic terms), insulin treatment (yes/no), and diabetic complications (yes/no).*

*Aβ1-42/Aβ1-40-Adjusted-2 means the Aβ ratio was adjusted for Model 1, plus rGSK-3β (Total/S9) (in continuous and quadratic terms), ApoE ε4 (yes/no), and olfactory score (in continuous).*

*Linear trend test using the median value of each category.

### Addition of the Elevated Plasma Aβ1-42/Aβ1-40 to Our Previous Model Enhanced the Diagnostic Efficacy of Mild Cognitive Impairment in Type 2 Diabetes Mellitus Patients

We have previously reported that an increasing aging, the increased olfactory score, the increased platelet GSK-3β activity, and the ApoE ε4 genotype were correlated with the cognitive decline in T2DM patients (Xu et al., 2016). By analyzing these four parameters, we received AUCs of 0.82 (95% CI: 0.76–0.87) and 0.86 (95% CI: 0.82–0.91), accuracy of 0.83 and 0.81, sensitivity of 0.58 and 0.71, and specificity of 0.91 and 0.86 for diagnosing cognitive decline in the training cohort and the validation cohort, respectively (Xu et al., 2016). Here, we first verified this previous model by the newly collected populations (*n* = 489) as a second validation. An AUC of 0.84 (95% CI: 0.81–0.87), accuracy of 0.78, sensitivity of 0.78, and specificity of 0.77 were generated (Supplementary Table 1). These results confirmed the repeatability of our previous model.

Then, we analyzed the efficacy by combining age, ApoE ε4, olfactory score, and platelet rGSK-3β data collected in the current study. AUCs of 0.846 (95% CI: 0.794-0.897) and 0.848 (95% CI: 0.815–0.882) and accuracy of 0.810 and 0.777 were generated, respectively, in the training cohort and the validation cohort. Importantly, we were able to obtain a significantly increased AUC in both the training cohort (0.869, 95% CI: 0.822–0.916) and the validation cohort (0.867, 95% CI: 0.835–0.899) when adding the elevated plasma Aβ1-42/Aβ1-40 to the above-mentioned four factors (Table 4). The C-statistics of the new model were significantly stronger than that of the previous model in the training cohort (AUC: 0.869 versus 0.846, *p* = 0.029) and in the validation cohort (AUC: 0.867 versus 0.848, *p* = 0.021) (Supplementary Table 2).

**TABLE 4:** Diagnosing efficacy of the combined biomarkers.

	Sensitivity	Specificity	Accuracy	AUC (95% CI)
**Training cohort**				
Age + ApoE ε4 + olfactory	0.674	0.792	0.763	0.776 (0.718–0.834)
Age + ApoE ε4 + olfactory + rGSK-3β	0.730	0.836	0.810	0.846 (0.794–0.897)
Age + ApoE ε4 + olfactory + rGSK-3β + Aβ1-42/Aβ1-40	0.764	0.869	0.843	0.869 (0.822–0.916)
**Validation cohort**				
Age + ApoE ε4 + olfactory	0.591	0.778	0.732	0.704 (0.657–0.750)
Age + ApoE ε4 + olfactory + rGSK-3β	0.716	0.849	0.777	0.848 (0.815–0.882)
Age + ApoE ε4 + olfactory + rGSK-3β + Aβ1-42/Aβ1-40	0.841	0.760	0.804	0.867 (0.835–0.899)

*Bootstrap resampling times = 1,000.*

*AUC, area under curve; CI, confidence interval.*

To further assess the improvements in diagnostic performance gained by adding new biomarkers to an existing risk diagnostic model, we used NRI. Compared with our previous model, our new model by addition of Aβ1-42/Aβ1-40 showed better discrimination with an NRI of 14.8% (95% CI: −1.6–32.9%) in the training cohort and 26.0% (95% CI: 6.7–46.5%) in the validation cohort. These data confirmed that addition of plasma Aβ1-42/Aβ1-40 could significantly improve the efficacy of the model in diagnosing MCI in T2DM patients.

### An Advanced New Model for Diagnosing Mild Cognitive Impairment in Type 2 Diabetes Mellitus Patients Established and Validated in the Training and Validation Cohorts

**FIGURE 2:**
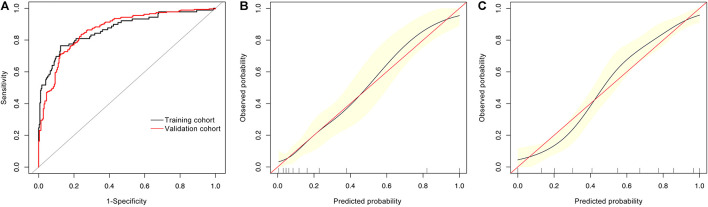
ROC and calibrating curves for diagnosing MCI in T2DM patients. By combining age, ApoE ε4 genotype, olfactory score, platelet rGSK-3β, and plasma Aβ1-42/Aβ1-40, we received AUCs of 0.869 (95% CI: 0.822–0.914) and 0.867 (95% CI: 0.835–0.899) in the training and validation cohorts, respectively **(A)** with high diagnosed probability [**(B)** the training cohort; **(C)** the validation cohort].

Finally, we did ROC analysis by combining Aβ1-42/Aβ1-40, rGSK-3β, ApoE ε4, olfactory score, and age in the training and validation cohorts (Figure 2A). The AUCs in the training and validation cohorts were 0.869 (95% CI: 0.822–0.916) and 0.867 (95% CI: 0.835–0.899), respectively. Accordingly, an advanced new model was generated: logit (MCI) = −9.87434 + 0.03576 × age + 0.58996 × (ApoE ε4 = 1) + 1.83487 × rGSK-3β(Total/S9) + 0.53539 × olfactory score + 1.01532 × Aβ1-42/Aβ1-40, which could be used to precisely discriminate MCI from T2DM patients. We further calibrated the accuracy of the new model, and the results showed that the 95% CIs of the calibration belt were perfectly fitted to the diagonal bisector line in both training and validation cohorts (Figures 2B,C). These data confirmed that the combination of age, ApoE ε4 genotype, olfactory score, platelet rGSK-3β, and plasma Aβ1-42/Aβ1-40 could diagnose MCI with high probability.

## Discussion

Early diagnosis of AD is difficult, which has seriously impeded its drug development and early intervention. T2DM patients have high incidence to suffer from AD (Li et al., 2015). With the increasing T2DM population, it is of great importance to find peripheral biomarkers to diagnose or eventually predict who will develop AD. In continuing with our previous multicenter case–control study (Xu et al., 2016), we found in the present study that Aβ1-42 in plasma was increased in T2DM-MCI compared with T2DM-nMCI patients, and the elevated ratio of Aβ1-42/Aβ1-40 could be an independent risk factor for MCI in T2DM patients. The addition of elevated plasma Aβ1-42/Aβ1-40 to our previous model (Xu et al., 2016) significantly enhanced the specificity, sensitivity, and accuracy in diagnosing MCI in T2DM patients. MMSE has been used in the clinic to test MCI. However, as a neuropsychiatric test, the diagnosis made by MMSE could be substantially influenced by the doctor’s subjective judgment. Additionally, the MMSE test has to be carried out in a clinician’s one-on-one setting, which is almost impossible for large-population screening, such as in community medical examination. For the same reason, the MMSE test has been currently not routinely applied in the clinic, including the endocrinology department. On the other hand, periphery biomarkers could be used to quickly and objectively screen MCI (or cognitive decline) in all clinical departments and community medical centers in high-risk populations.

Measurements of Aβ42 in CSF by ELISA and amyloid in the brain by PET imaging are increasingly integrated into the clinical workup of AD as biomarkers of amyloid pathology. The changes of Aβ1-42 and Aβ1-42/Aβ1-40 levels in CSF are correlated with the changes of Aβ in the plasma of both control and MCI populations (Janelidze et al., 2016). However, contradictory results have been observed regarding the changes of Aβ in CSF and blood. Some reported that a high level of plasma Aβ1-42 or an increased Aβ1-42/Aβ1-40 ratio was a risk factor for future AD, while others reported the opposite data (Mayeux et al., 1999, 2003; Irizarry, 2004; Pomara et al., 2005; van Oijen et al., 2006; Graff-Radford et al., 2007; Blennow et al., 2010; Cosentino et al., 2010; Buchhave et al., 2012). The reason for the discrepancy is currently not understood. By a matched case–control study, it was found that the Aβ1-42/Aβ1-40 ratio was significantly increased in T2DM patients compared with that in the non-T2DM controls (Peters et al., 2017). To our best knowledge, the present study is the first to establish a positive association between the increased plasma Aβ1-42/Aβ1-40 ratio and mild cognitive decline among T2DM patients. The mechanism by which plasma Aβ establishes the connection with CSF is poorly understood. The brain biopsy analyses suggest that the blood–brain barrier (BBB) dysfunction is a characteristic feature of AD (Claudio, 1996), which may cause leakage of brain Aβ into the peripheral blood. The peripheral Aβ can also enter the impaired BBB and thus cause neurovascular unit impairments during disease progression (Yamazaki and Kanekiyo, 2017). We speculate that the increased plasma Aβ may enter the brain through the damaged BBB and deposit as senile plaques, thereby inducing MCI during the progression of T2DM.

We also found that the MCI in T2DM patients had a strong association with platelet GSK-3β activation and that GSK-3β could also serve as an independent risk factor for MCI in T2DM patients. As an important kinase located in the insulin pathway, GSK-3β links T2DM and AD pathologies (Zhang et al., 2018). Upregulation of GSK-3β induces Aβ production and tau hyperphosphorylation, leading to memory impairment (Hooper et al., 2008; Zhang et al., 2016). In patients with early AD, intranasal administration of insulin to inhibit GSK-3 activity successfully increases the ratio of Aβ40/42 with improved cognition (Reger et al., 2008). As GSK-3β has broad substrates and biological functions, targeting GSK-3β causes significant side effects. However, measuring the upregulated peripheral GSK-3β by a simple and well-reproducible protocol (Xu et al., 2016) provided a convenient and low-cost tool for diagnosing MCI in T2DM patients. As we are currently unable to have brain or CSF samples from these T2DM patients, we are unfortunately not able to identify whether the platelet GSK-3β activation reflects GSK-3β activation in the central nervous system or to provide direct evidence revealing the possible biological relationship between platelet GSK-3β and cognitive impairment. Nonetheless, platelets are frequently used to search for peripheral biomarkers for the nervous system because they share many biochemical similarities, such as accumulation and release of neurotransmitters, responses to variations of calcium concentration, and expression of receptors and enzymes, and therefore, the platelet has served as an important model for the investigation of metabolic abnormalities in AD (Zubenko et al., 1999; Gattaz et al., 2004; Forlenza et al., 2011). How exactly the platelet GSK-3β activation may reflect brain GSK-3β activation and its biological relations with cognitive impairment deserve further investigation.

Additionally, it is well known that aging is the most fundamental factor contributing to AD. Postmortem studies indicate that AD neuropathology emerges early in areas involved in olfactory information processing before the onset of clinical symptoms (Braak and Del Tredici, 2011). ApoE ε4 is the most prevalent understudied risk factor for AD (Michaelson, 2014). Therefore, an established model incorporating aging, ApoE ε4 genotype, olfactory decline, an increased plasma Aβ1-42/Aβ1-40, and an increased platelet GSK-3β activity has a great potential for predicting who may develop dementia (including AD) in T2DM patients. Our results are cross-centered and longitudinally validated to provide reliability for the observed association.

The limitations for the current study are as follows. First, the case–control nature of the study could not establish the causal role of plasma Aβ1-42/Aβ1-40 and other factors in the pathogenesis of MCI; thus, further prospective studies are needed. Second, the study did not evaluate the islet amyloid polypeptide (Janson et al., 2004), which could also be associated with cognitive impairment. Third, the participants recruited in the study were middle-aged or elderly Han Chinese, which limits generalization to other ages or races. However, the homogeneity of age and ethnicity may reduce residual confounders. Fourth, considering the semi-quantitative nature of Western and dot blotting analyses, future studies should try ELISA to quantitatively evaluate GSK-3β activity.

In summary, we provide the first evidence in the present study that the increased ratio of plasma Aβ1-42/Aβ1-40 is an independent risk factor for cognitive decline in T2DM patients. By adding the elevated Aβ1-42/Aβ1-40 to our previous model, we establish a new model with higher specificity, sensitivity, and accuracy in discriminating MCI from non-MCI in T2DM patients. The model is applicable for early diagnosis and timely intervention of MCI in a rapidly increasing number of T2DM patients, which may eventually decrease the prevalence of AD.

## Data Availability Statement

The original contributions presented in the study are included in the article/Supplementary Material, further inquiries can be directed to the corresponding author.

## Ethics Statement

The studies involving human participants were reviewed and approved by Medical Ethics Committee, Tongji Medical College, Huazhong University of Science and Technology. The patients/participants provided their written informed consent to participate in this study.

## Author Contributions

J-ZW and YCL designed the research and wrote the manuscript. YCL, SJZ, BH, SZ, YZ, WW, ZPX, XW, ZHX, YY, YG, DK, SL, WJW, HY, DW, GP, TJ, MD, RX, HL, NT, QZ, JY, ML, NH, GS, WP, CZ, WM, YNL, and HZ recruited the patients and collected blood samples. YCL and BH performed the experiments. YCL and LC performed the statistical analysis. LC contributed to data interpretation and manuscript editing. J-ZW was the guarantor of this work and has full access to all the data in the study, and took responsibility for the integrity of the data and accuracy of the data analysis. All authors read and approved the final manuscript.

## Conflict of Interest

The authors declare that the research was conducted in the absence of any commercial or financial relationships that could be construed as a potential conflict of interest.

## Publisher’s Note

All claims expressed in this article are solely those of the authors and do not necessarily represent those of their affiliated organizations, or those of the publisher, the editors and the reviewers. Any product that may be evaluated in this article, or claim that may be made by its manufacturer, is not guaranteed or endorsed by the publisher.

## Footnotes

1
http://www.r-project.org

2
http://www.empowerstats.com
