# Supplementary material for: Periphery Biomarkers for Objective Diagnosis of Cognitive Decline in Type 2 Diabetes Patients
Source: Front Cell Dev Biol. 2021 Oct 20;9:752753. doi: 10.3389/fcell.2021.752753 (PMC8564071; doi:10.3389/fcell.2021.752753)
Supplement: Supplementary file 1 [file Data_Sheet_1.docx]

**SUPPLEMENTARY DATA**

**Supplementary Table 1. The second validation of our previous model for MCI prediction in T2DM patients**

| Variables | Sensitivity | Specificity | Accuracy | AUC (95% CI) |
| --- | --- | --- | --- | --- |
| The training set (our previous study) [1] |  |  |  |  |
| Age + ApoE ε4 + olfactory + rGSK3β | 0.58 | 0.91 | 0.83 | 0.82(0.76-0.87) |
|  |  |  |  |  |
| The validation set (our previous study) [1] |  |  |  |  |
| Age + ApoE ε4 + olfactory + rGSK3β | 0.71 | 0.86 | 0.81 | 0.86(0.82-0.91) |
|  |  |  |  |  |
| The validation set (the present study) |  |  |  |  |
| Age + ApoE ε4 + olfactory + rGSK3β | 0.78 | 0.77 | 0.78 | 0.84(0.81-0.87) |

[1] Xu ZP, Yang SL, Zhao S, Zheng CH, Li HH, Zhang Y, et al. Biomarkers for Early Diagnostic of Mild Cognitive Impairment in Type-2 Diabetes Patients: A Multicentre, Retrospective, Nested Case-Control Study. EBioMedicine. 2016; 5: 105-13.

**Supplementary Table 2. Comparison of the discrimination between these two models.**

|  | Old Model (Training set) | New Model (Training set) | *P* (compare) | Old Model (Validation set) | New Model (Validation set) | *P* (compare) |
| --- | --- | --- | --- | --- | --- | --- |
| T2DM-MCI, *n* | 89 | 89 |  | 264 | 264 |  |
| T2DM-nMCI, *n* | 274 | 274 |  | 225 | 225 |  |
| C-index (AUC) | 0.8456 | 0.869 | 0.029 | 0.8482 | 0.8671 | 0.021 |
| 95%CI lower | 0.7939 | 0.8215 |  | 0.8145 | 0.8353 |  |
| 95%CI upper | 0.8973 | 0.9164 |  | 0.8818 | 0.8989 |  |
| Best threshold | -1.1704 | -1.1275 |  | -0.6513 | -1.3187 |  |
| Specificity | 0.8358 | 0.8686 |  | 0.8489 | 0.76 |  |
| Sensitivity | 0.7303 | 0.764 |  | 0.7159 | 0.8409 |  |
| Accuracy | 0.8099 | 0.843 |  | 0.7771 | 0.8037 |  |

Bootstrap resampling times = 1000

Old Model: logit (MCI) = -8.23288 + 0.04055 × Age + 2.22926 × rGSK-3β (Total/S9) + 0.58349 × (ApoE ε4=1) + 0.45079 × Olfactory score

New Model: logit (MCI) = -9.02221 + 0.04348 × Age + 2.21718 × rGSK-3β (Total/S9) + 0.66016 × (ApoE ε4=1) + 0.46446 × Olfactory score + 0.82053 × Aβ42/40
